# Metformin Promotes Differentiation and Attenuates H_2_O_2_-Induced Oxidative Damage of Osteoblasts *via* the PI3K/AKT/Nrf2/HO-1 Pathway

**DOI:** 10.3389/fphar.2022.829830

**Published:** 2022-03-21

**Authors:** Keda Yang, Fangming Cao, Shui Qiu, Wen Jiang, Lin Tao, Yue Zhu

**Affiliations:** Department of Orthopedics, First Hospital of China Medical University, Shenyang, China

**Keywords:** metformin, osteogenic differentiation, RNA sequencing, oxidative damage, PI3K/AKT/Nrf2/HO-1

## Abstract

At present, the drug treatment of osteoporosis is mostly focused on inhibiting osteoclastogenesis, which has relatively poor effects. Metformin is a drug that can potentially promote osteogenic differentiation and improve bone mass in postmenopausal women. We aimed to detect the molecular mechanism underlying the osteogenic effect of metformin. Our study indicated that metformin obviously increased the Alkaline phosphatase activity and expression of osteogenic marker genes at the mRNA and protein levels. The PI3K/AKT signaling pathway was revealed to play an essential role in the metformin-induced osteogenic process, as shown by RNA sequencing. We added LY294002 to inhibit the PI3K/AKT pathway, and the results indicated that the osteogenic effect of metformin was also blocked. Additionally, the sequencing data also indicated oxidation-reduction reaction was involved in the osteogenic process of osteoblasts. We used H_2_O_2_ to mimic the oxidative damage of osteoblasts, but metformin could attenuate it. Antioxidative Nrf2/HO-1 pathway, regarded as the downstream of PI3K/AKT pathway, was modulated by metformin in the protective process. We also revealed that metformin could improve bone mass and oxidative level of OVX mice. In conclusion, our study revealed that metformin promoted osteogenic differentiation and H_2_O_2_-induced oxidative damage of osteoblasts *via* the PI3K/AKT/Nrf2/HO-1 pathway.

## Introduction

Osteoporosis is characterized by a decrease in the amount of bone tissue per unit volume and mainly occurs in postmenopausal women and diabetes patients (Chapurlat et al., 2020; Zhou et al., 2020). Estrogen deficiency and glucose overload reduce the inhibition of osteoclast differentiation and enhances bone resorption, leading to the loss of bone mass (Ponte et al., 2020; Cho et al., 2021; Sha et al., 2021). The current strategy for osteoporosis treatment is mainly to inhibit osteoclasts with drugs such as bisphosphonates, estrogen receptor modulators and calcitonin (Ukon et al., 2019; Hsiao et al., 2020; Toriumi et al., 2020). However, these drugs are limited because they only prevent further loss of bone mass, and do not restore it. To improve this condition, researchers can increase the activity of osteoblasts in patients, which will be an effective means of treatment. Weakened differentiation and declined activity of osteoblasts aggravate the deterioration of osteoporosis (Xu et al., 2020; Yang et al., 2020). Therefore, enhancing differentiation and increasing activity is essential in improving the treatment of osteoporosis.

The acceleration of the aging population has resulted in a gradual increase in the incidence of osteoporotic fractures. Estrogen deficiency and impaired glucose tolerance are important factors that accelerate aging (He et al., 2020; Kashima et al., 2021). And the decline in body function related to aging is caused by oxidative damage with the accumulation of reactive oxygen species (ROS) (Buccellato et al., 2021). Recent studies have determined that metformin shows anti-aging effects and resistance to diseases caused by aging, which indicates the potential application of metformin in osteoporosis (Martel et al., 2021; Sharma et al., 2021). Metformin is a hypoglycemic drug that has been verified to improve bone mass loss in diabetes-induced osteoporosis by decreasing the blood glucose level (Tseng, 2021). And metformin also shows potential therapeutic effects in postmenopausal osteoporosis (Yang et al., 2021). However, the direct effect of metformin on bone metabolism is unclear. The molecular mechanism of metformin on osteogenic differentiation and prevention of oxidative damage remain to be determined.

A new generation of high-throughput transcriptome sequencing methods have been used in basic research, clinical diagnostics and drug development (Koborova et al., 2009; Wang et al., 2013). RNA sequencing technology has become an important method in transcriptomic research. The principle is to sequence genomic cDNA, calculate the expression of different mRNAs by counting the number of related small cDNA fragments and analyze the expression level of the transcript (Niemi et al., 2015). This technology can elucidate gene function and structure at the overall level and reveal specific biological processes and molecular mechanisms in the occurrence of diseases (Cui et al., 2020; Wang Y et al., 2021). In previous studies, RNA sequencing has been widely used to determine the targets and pathways of drugs in disease research (Chen et al., 2021; Zhou et al., 2021). Therefore, we aimed to detect the molecular mechanism of metformin in osteogenic differentiation by using RNA sequencing.

## Materials and Methods

### Reagents, Cell Culture and Osteogenic Differentiation

The reagents and chemicals used in this study are listed below. MC3T3-E1 cells were purchased from the Chinese Academy of Sciences Cell Bank. Metformin was purchased from Meilunbio (Dalian, China). Antibodies against Runx2 (1:1000; cat. no. ab236639), Collagen Ⅰ (1:2500; cat. no. ab260043) Osteocalcin (OCN) (1:100; cat. no. ab93876), Nrf2 (1:1000, cat. no. ab92946) and HO-1 (1:5000; cat. no. ab68477) were obtained from Abcam (Cambridge, MA). β-actin antibodies (1:2000; cat. no. 66009-1-Ig) and a peroxidase-conjugated anti-rat secondary antibody (1:2000; cat. nos. SA00001-15) were purchased from Protein Tech Group, Inc. (Chicago, IL, United States). antibodies against PI3K (1:1,000; cat. no. 4257), phosphorylated (p-)PI3K (1:1,000; cat. no. 4228), AKT (1:1,000; cat. no. 4691) and p-AKT (1:1,000; cat. no. 4060) were purchased from Cell Signaling Technology, Inc. The PI3K/AKT signaling inhibitor LY294002 and AKT inhibitor MK2206 was purchased from Beyotime (Shanghai, China).

MC3T3-E1 cells were cultured in α-MEM (HyClone, Logan, UT, United States). The media were supplemented with 10% fetal bovine serum, 100 U/ml streptomycin sulfate, and 100 mg/ml penicillin. Osteogenic induction medium was prepared according to the following criterion: 100 mM β-glycerophosphate, 50 mg/L ascorbic acid and 10 nM dexamethasone. The parameters of the humidified incubator for MC3T3-E1 cell culture were set to 5% CO_2_ and 37°C. Detection of ALP activity and mRNA and protein expression levels was performed after 7 days of osteogenic induction of MC3T3-E1 cells.

### Cell Counting Kit-8 Assay

Cell Counting Kit-8 (CCK-8) (Dojindo Molecular Technologies, Inc. Japan) was used to detect cell viability after treatment with different concentrations of metformin. The reagent is an indicator of redox reactions. In the presence of the electron carrier 1-methoxy PMS, dehydrogenase in living cells can catalyze the tetrazolium salt WST-8 to generate formazan dyes, and the amount of formazan dye produced has a linear relationship with the number of living cells.

### Alkaline Phosphatase Activity Detection

ALP is secreted by osteoblasts. ALP activity can directly reflect the differentiation level of osteoblasts. MC3T3-E1 cells were induced in osteogenic medium for 1 week. Then the ALP level was detected by ALP Analysis kits (Nanjing Jiancheng, China) according to the manufacturer’s instructions.

### Alizarin Red S Staining

Calcium salt variation is an indicator of osteoblast proliferation and differentiation. Alizarin Red S can form a complex with calcium salt in a chelating manner to identify the calcium salt component of tissue cells and produce orange-red deposits. MC3T3-E1 cells were cultured in 6-well plates and treated with induced medium for 28 days before Alizarin red S staining. The cells were first washed twice with PBS, fixed with 95% ethanol for 10 min, and washed with distilled water 3 times. Then 0.1% Alizarin Red-Tris-Hcl (pH 8.3) was added at 37°C for 30 min.

### Reverse Transcription PCR (RT-qPCR) Assay

A miRNeasy RNA mini kit (Qiagen, MD, United States) was used to extract total RNA. Then, GoScript™ reverse transcription mix and oligo (dT) (Promega, Wi, United States) were used to synthesize cDNA. qPCR was performed using GoTaq® qPCR master mix (Promega, Wi, United States). The data were collected using a Roche Light Cycler® 480 II system (Roche, Basel, Switzerland). The conditions of PCR cycles were as follows: 2 min at 90°C, 15 s at 95°C and 60 s at 60°C for 45 cycles. *β*-actin was used as a standardized control. And the sequences of primers were listed in Supplementary Table S1. Gene expression was calculated by the 2^−ΔΔCt^ method.

### Western Blotting

Protein was extracted after 7 days of treatment, and then frozen in a refrigerator at −80°C for later use.

Then, proteins were resolved by SDS-PAGE and transferred to polyvinylidene difluoride (PVDF) membranes. The membranes with proteins of various molecular weights were immersed in blocking buffer for 1.5 h. After the membranes were washed with 1% TBST, they were incubated with a primary antibody at 4°C overnight and a secondary antibody at 4°C the next day. After the membranes were thoroughly washed, the protein bands were coated with luminescent solution and visualized using a chemiluminescence (ECL) system (UVP Inc., CA, United States). The protein level was normalized to that of *β*-actin (molecular weight of 43 kDa). Finally, ImageJ software was used to calculate the optical density and relative protein expression levels.

### RNA Sequencing Analysis

MC3T3-E1 cells were induced in osteogenic medium and metformin for 3 days. RNA sequencing analysis was performed by Novogene Institute (Tianjin, China). Differential expression analysis of two groups (three biological replicates per condition) was performed using the DESeq2 R package (1.20.0). Gene Ontology (GO) enrichment analysis of differentially expressed genes was implemented by the cluster Profiler R package, in which gene length bias was corrected. GO terms with corrected *p* values less than 0.05 were considered significantly enriched by differentially expressed genes. The KEGG pathway database was used in the cluster Profiler R package for the statistical enrichment of marker genes.

### Gene Ontology Enrichment

GO enrichment analysis of marker genes was performed with DAVID Bioinformatics Resources 6.8. The gene length bias was corrected, and the GO terms with corrected *p* values less than 0.05 were considered significantly enriched for the marker genes. The biological domains examined in the GO database included three categories: molecular function, cellular component and biological process.

### KEGG Pathway

The KEGG pathway database (KOBAS 3.0) was used for the statistical enrichment of marker genes. The database can help elucidate the high-level functions and utilities of cells, organisms and ecosystems and includes molecular-level information (especially large-scale molecular genome sequencing datasets) and information from other high-throughput experimental technologies.

### Animal Experiments

Eight-week-old female mice were obtained from the Department of Laboratory Animal Science of China Medical University. The feeding environmental conditions were 20–26°C with constant temperature, 40–70% relative humidity, ≤14 mg/m3 ammonia concentration, ≤60 dB (A) noise and a 12 h/12 h alternating light and dark cycle. All animals were fed in this environment for 2 weeks before the experiments. Mice were randomly divided into three groups (*n* = 7 each group): a sham group, a bilateral ovariectomy (OVX) group and a OVX group with intragastric metformin (OVX + Met). We performed bilateral ovariectomy on mice under 1.4–1.5% isoflurane inhalation anesthesia with oxygen. The dosage of metformin for the OVX + Met mice was 100 mg/kg/day, which was dissolved in 0.9% normal saline. The mice in the other groups were fed only with equal amount of saline. After 8 weeks of treatment, all mice were sacrificed, and the bilateral femur and tibias were harvested for imaging and protein extraction. All animal experiments were approved by the Animal Ethics Committee of the First Affiliated Hospital of China Medical University and were performed according to the laboratory and animal welfare guidelines.

### Microcomputed Tomography

Collected femurs were fixed with 4% formaldehyde solution for imaging by microcomputed tomography (μCT, Skyscan1276, Bruker, Germany). When X-rays pass through the sample, each part of the sample has different absorption rates for X-rays. The X-rays penetrate the sample and are finally imaged on the detector. Micro-CT uses tapered X-ray beams to image samples at different angles above 360°. The cone beam method can obtain isotropic volumetric images, improve spatial resolution, and increase ray utilization. X-ray images at each angle were reconstructed into a 3-dimensional image analyzed by the CT-analyser software CTAn 1.19.11.1 (Bruker Corporation). The parameters of the scan were as follows: X-ray voltage 50 kV, X-ray current 200 uA, Filter 0.5 mm aluminum, Image pixel size 8.9um, Camera resolution setting High (4,000 pixel field width), Tomographic rotation (180°/360°), Rotation step (0.3–0.5°), Frame averaging 1, Scan duration 20–50 min.

### Biochemistry Assays

Mice-specific total antioxidant activities (T-AOC) was purchased from Nanjing Jiancheng Bioengineering Institute, Nanjing, China (A015-2-1) and superoxide dismutase (SOD) kits was purchased from Beyotime (S0101) to measure the serum T-AOC and SOD activity in mice. All experiments were performed according to the manufacturer’s instructions.

### Statistical Analysis

The experimental data were means ± standard deviation (SD) by using GraphPad Prism 8 (San Diego, CA, United States) and SPSS 22.0 (Chicago, IL, United States). Student’s t-tests and one-way ANOVA were used for statistical analysis of three replicate experiments by SPSS. *p* < 0.05 was considered statistically significant.

## Results

### Metformin Promotes Osteogenic Differentiation of MC3T3-E1 Cells

**FIGURE 1:**
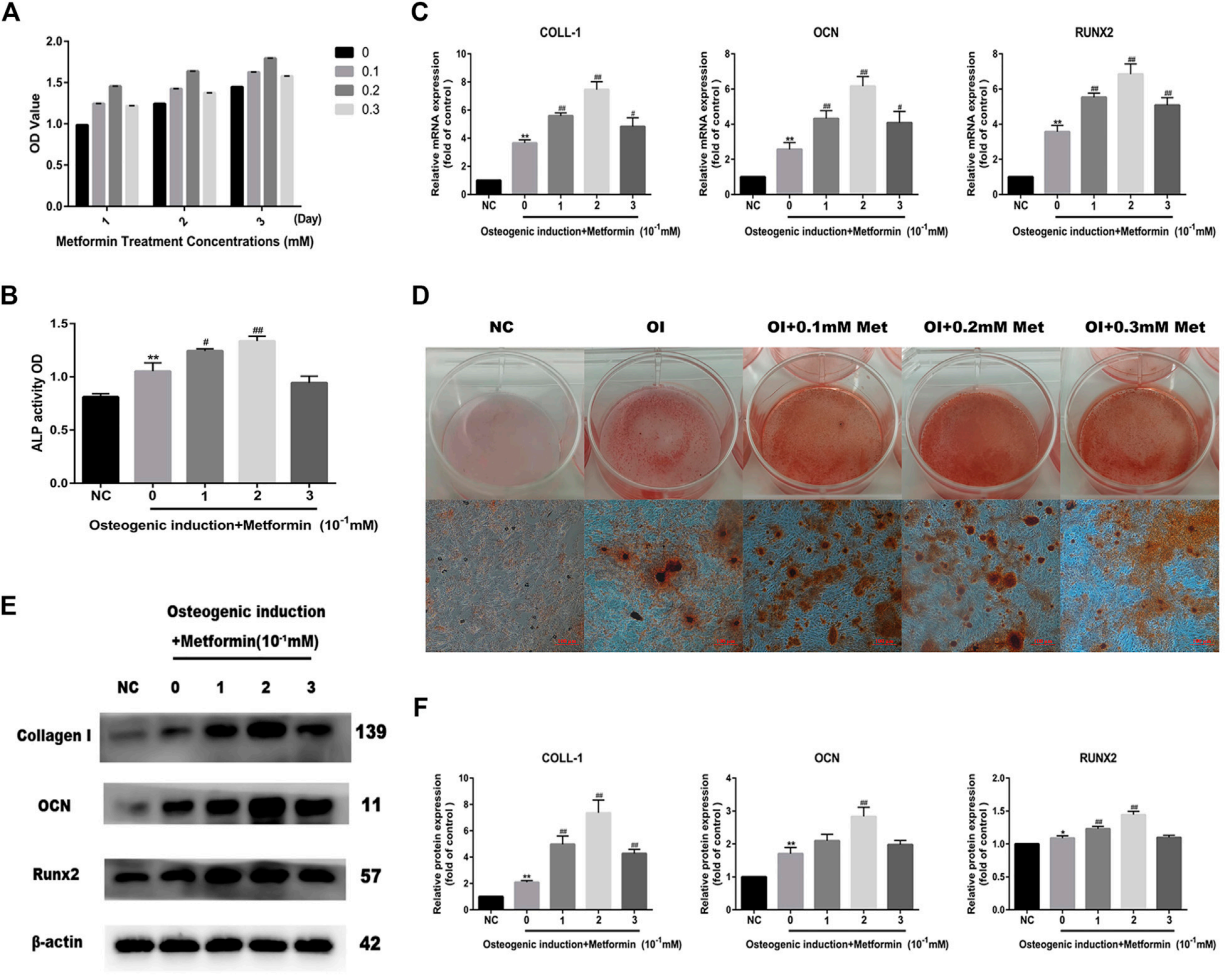
Metformin promotes osteogenic differentiation of MC3T3-E1 cells. **(A)** Cell viability after treatment with different concentrations of metformin (0.1, 0.2 and 0.3 mM). **(B)** ALP activity was detected to explicit the osteogenic effect of metformin. **(C)** The mRNA level of COLL-1, OCN and Runx2 after osteogenic induction and treatment with metformin. **(D)** The mineralization of osteoblast demonstrated by Alizarin red S staining was most obvious after treatment with 0.2 mM metformin. **(E)** The protein level of Collagen I, OCN and Runx2 under different concentrations of metformin. **(F)** Relative protein expression level of the proteins in **(E)** compared with control group. Experiments were implemented in triplicate. Data are means ± SD, *p < 0.05, **p < 0.01 compared with control cells and ^#^ *p* < 0.05, ^##^ *p* < 0.01 compared with osteogenic induction alone analyzed by using ANOVA.

We first performed CCK-8 cell viability assays to detect MC3T3-E1 cell viability after treatment with different concentrations of metformin, and the results indicated that metformin had no inhibitory effect on osteoblast proliferation (Figure 1A). To determine whether metformin promoted osteogenic differentiation and the optimal concentration, we detected ALP activity with ALP Analysis kits. As shown in Figure 1B, metformin at 0.2 mM maximized osteogenic differentiation, which was also verified by the mRNA expression of osteogenic genes including Runx2, OCN and COLL-1 (Figure 1C). Additionally, we performed Alizarin Red S staining and western blotting to evaluate the effect of metformin treatment on the differentiation of osteoblasts (Figures 1D,E). The protein levels of Collagen I, Runx2 and OCN increased most obviously with 0.2 mM metformin treatment (Figure 1F).

### RNA-Sequencing Reveals That PI3K/AKT Signaling Pathway is involved in Metformin-Induced Osteogenic Differentiation

**FIGURE 2:**
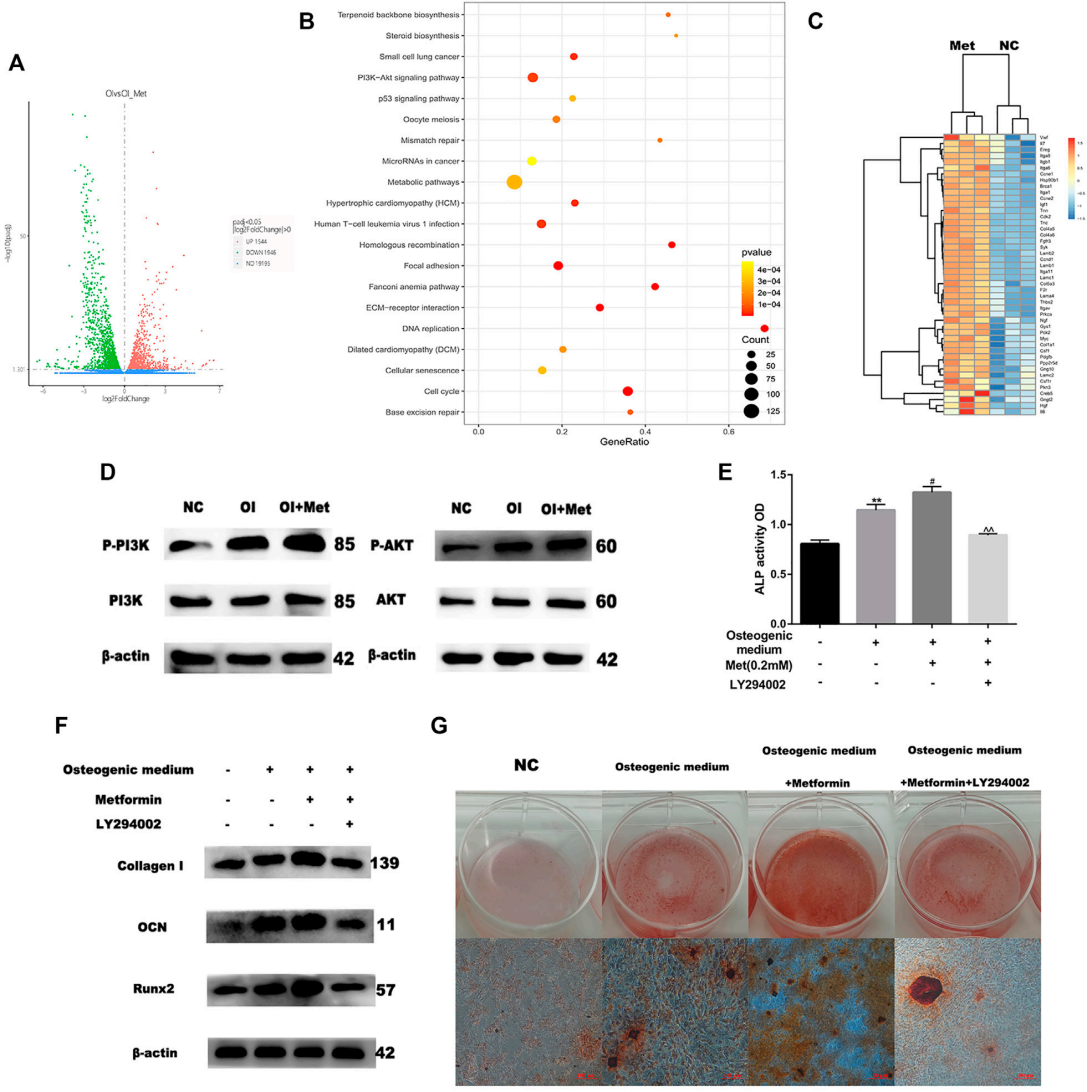
Metformin induces osteogenic differentiation *via* the PI3K/AKT pathway. **(A)** Volcano map of differential genes as osteogenic induction (OI) vs. OI with metformin treatment (OI_Met). **(B)** Kyoto Encyclopedia of Genes and Genomes (KEGG) of up-regulated genes during metformin-induced osteogenic differentiation. **(C)** Heatmap of up-regulated genes involved in PI3K/AKT signaling pathway. **(D)** Protein expression of PI3K-AKT pathway, enhancing effect observed for metformin on PI3K/AKT expression. **(E)** ALP activity was detected to explicit metformin protection of osteogenic differentiation inhibited by H_2_O_2_ was blocked by LY294002. **(F)** Protein expression of COLL-1, OCN and Runx2 after adding LY294002. **(G)** The activity of osteoblast mineralization decreased with LY294002 treatment detected by using Alizarin red S staining. Experiments were implemented in triplicate. Data are means ± SD, *p < 0.05, **p < 0.01 compared with control cells and ^#^ *p* < 0.05, ^##^ *p* < 0.01 compared with osteogenic induction alone and ^*p* < 0.05, ^^*p* < 0.01 compared with combined treatment of osteogenic induction and metformin analyzed by using ANOVA.

Based on the data mentioned above, we attempted to elucidate the mechanism by which metformin promoted osteoblast differentiation. We performed RNA sequencing to compare the differential expression of genes between osteogenic induction with and without 0.2 mM metformin treatment. Compared with osteogenic medium alone, a total of 1946 up-regulated and 1544 down-regulated genes were obtained when treating with metformin after quality control and differential analysis (Figure 2A). We performed KEGG pathway analysis of these up-regulated genes and the results showed that they were enriched in PI3K/AKT signaling pathway (Figure 2B). These genes included PI3K upstream regulators ITGA, ITGB and SYK; the AKT upstream regulators HSP90B1 and PPP2R5D; and the downstream regulators of the PI3K/AKT pathway GYS, PCK2 and CCND1 (Figure 2C). We firstly detected the effect of metformin on the PI3K/AKT pathway by western blotting. And the results indicated the expression of p-PI3K and p-AKT increased in osteogenic medium and metformin promoted it further (Figure 2D). To determine the role of PI3K/AKT signaling pathway in osteogenic induction by metformin, we added the PI3K/AKT signaling inhibitor LY294002 to MC3T3-E1 cells and assessed osteogenic indicators. LY294002 can inhibit the enzyme activity of PI3K through competitive inhibition of PI3K. In Figure 2E, ALP activity decreased after combined treatment with metformin and LY294002 compared to that with metformin treatment alone. Moreover, the protein levels of Collagen I, OCN and Runx2 decreased after combined treatment with metformin and LY294002 compared with metformin treatment alone (Figure 2F). Additionally, we performed Alizarin Red S staining to confirm the effect of the PI3K/AKT signaling pathway on osteoblast formation. The results indicated that LY294002 inhibited mineralization after osteogenic induction (Figure 2G).

### Metformin Prevents the Oxidative Damage of Osteoblasts Induced by H_2_O_2_ *via* the AKT/Nrf2/HO-1 Pathway

**FIGURE 3:**
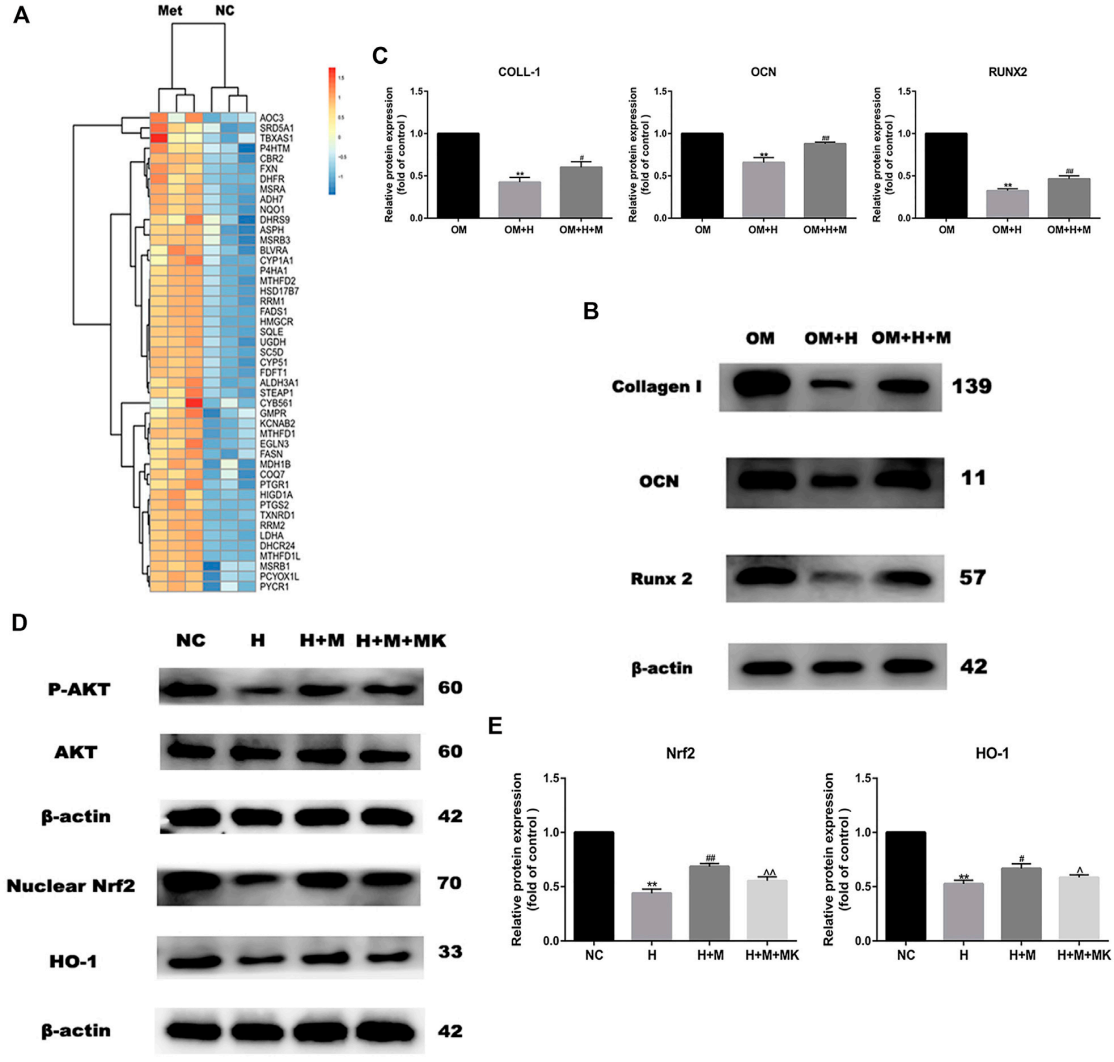
Metformin reserves H_2_O_2_-induced inhibition of osteogenic differentiation. **(A)** Heatmap of up-regulated genes involved in oxidation-reduction reaction. **(B)** Protein expression of Collagen I, Runx 2 and OCN indicated the protective effect of metformin on H_2_O_2_ inhibition (OM: osteogenic medium, OM + H: osteogenic medium with H_2_O_2_, OM + H + M: osteogenic medium mixed with H_2_O_2_ and MK2206). **(C)** Relative protein expression level of the proteins in **(B)**. **(D)** Protein expression of p-AKT, nuclear Nrf2 and HO-1 after additing AKT inhibitor MK2206. **(E)** Relative protein expression level of the proteins in **(D)**. Experiments were implemented in triplicate. Data are means ± SD, *p < 0.05, **p < 0.01 compared with control cells and ^#^ *p* < 0.05, ^##^ *p* < 0.01 compared with H_2_O_2_ treatment alone and ^*p* < 0.05, ^^*p* < 0.01 compared with combined treatment of H_2_O_2_ and metformin analyzed by using ANOVA.

GO analysis revealed that the genes upregulated expression after metformin treatment were involved in the oxidation-reduction reaction (Figure 3A). These genes included many reductase genes such as CBR2, HMGCR, HSD17B7, RRM2, DHCR24, DHFR, and peroxidase gene PTGS2. All of these genes performed a reductive effect on oxidative substances. In order to detect the protective effect of metformin on osteogenic differentiation in peroxidative status, we added 0.2 mM H_2_O_2_ into osteogenic medium to stimulate oxidative damage and 0.2 mM metformin to improve. After osteogenic induction and 6 h incubation with H_2_O_2_ and metformin, we detected the protein expression of Collagen I, OCN and Runx2 (Figure 3B). The results indicated the protective effect of metformin (Figure 3C). Then, we attempted to explore the mechanism by which metformin protected the oxidative damage of osteoblasts. Our past studies have proved that metformin can attenuate H_2_O_2_-induced osteoblast apoptosis *via* the PI3K/AKT pathway (Yang et al., 2021). Nrf2 is an important transcription factor that regulates cellular oxidative stress response. And the Heme Oxygenase-1 (HO-1) is the downstream factor of Nrf2. HO-1 participated in the modulation of mitochondria function and further influenced cell differentiation and apoptosis. We used western blotting to detect the protein level of nuclear Nrf2 and HO-1. Metformin increased the expression of Nrf2 and HO-1 compared with H_2_O_2_ treatment alone, but which was blocked by adding AKT inhibitor MK2206(Figures 3D,E).

### Metformin Prevents Bone Mass Loss and Reserves Oxidative Damage in OVX Mice

**FIGURE 4:**
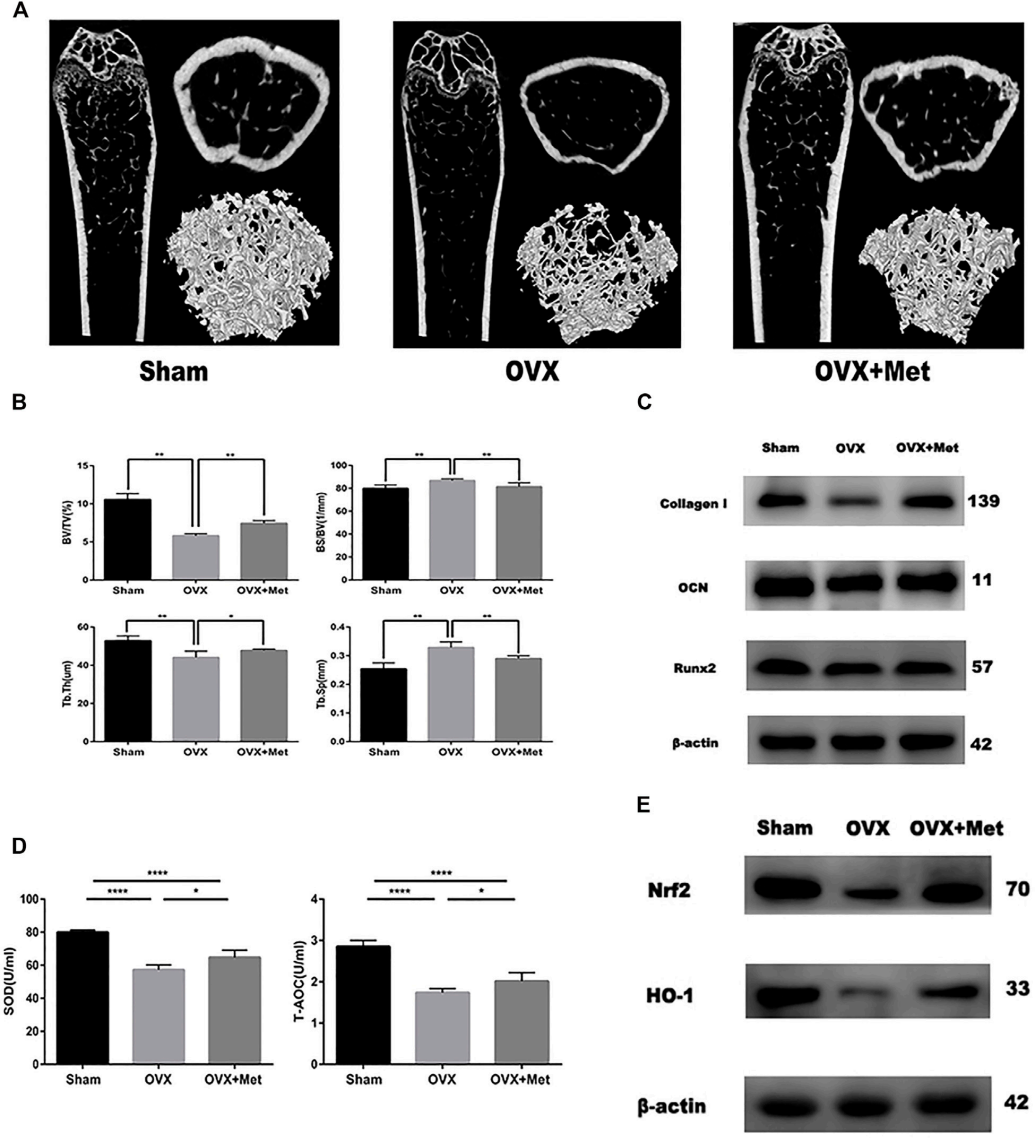
Metformin reserved bone mass loss of OVX mice. **(A)** 2D and 3D reconstruction of the femur micro-CT images. **(B)** Related parameters obtained from image analysis (n = 7 specimens/group). **(C)** Protein level of osteogenic markers Collagen I, Runx2 and OCN in femur samples. **(D)** Serum anti-oxidative level SOD1 and T-AOC in mice (*n* = 5 specimens/group). **(E)** The protein expression of Nrf2 and HO-1 in bone tissue. Data are means ± SD, *p < 0.05, **p < 0.01, ***p < 0.001, ****p < 0.0001 compared with OVX analyzed by using ANOVA.

Finally, we attempted to determine whether metformin could improve bone mass and oxidative level in postmenopausal mice. We performed bilateral ovariectomy on mice to decrease estrogen secretion and fed them a normal diet. Additionally, we fed another set of OVX mice metformin. After 8 weeks, the bone mass of the OVX mice decreased significantly but increased after metformin feeding (Figure 4A). The micro-CT data also revealed the therapeutic effect of metformin on postmenopausal osteoporosis. Trabecular thickness (Tb.Th) and percent bone volume (bone volume/tissue volume, BV/TV) decreased in the OVX mice and increased with metformin treatment. Trabecular separation (Tb.Sp) and bone surface/volume ratio (bone surface/bone volume, BS/BV) increased in the OVX mice and metformin preserved these parameters (Figure 4B). To detect the osteogenic effect of metformin on postmenopausal mice, we also extracted protein from the mouse femur. As shown in Figure 4C, the osteogenic proteins Collagen I, OCN and Runx2 were decreased compared with those in the sham mice, and metformin improved their expression levels. And we also collected the blood of these mice and centrifuged it into serum. The level of superoxide dismutase (SOD) and total antioxidant capacity (T-AOC) were measured by the corresponding kit. And the results indicated metformin reserved the serum level of SOD and T-AOC (Figure 4D). We measured the protein expression of Nrf2 and HO-1 to validate the signaling pathway in bone tissue (Figure 4E). As mentioned above, metformin could improve bone mass loss by enhancing the osteogenic effects and reserving oxidative damage in the OVX mice.

## Discussion

With increased global aging, osteoporosis is a major threat to human health and quality of life, and postmenopausal women have the greatest risk (Chen et al., 2019). The incidence of osteoporotic fractures, which increases the health care burden, is increasing (Jørgensen et al., 2017; Kanis et al., 2021). In recent years, metformin has been found to be a multipotent anti-aging drug with cardiovascular protective and tumor growth inhibitory effects (Bahrambeigi et al., 2019; Elgendy et al., 2019; Deshmukh et al., 2021; Kodali et al., 2021). However, previous studies focused on the hypoglycemic effect of metformin in diabetes-induced osteoporosis but ignored the direct effect on bone metabolism and the molecular mechanism underlying the osteogenic differentiation of metformin. Our study determined the osteogenic effect and optimal concentration of metformin on MC3T3-E1 cell differentiation and the therapeutic effect on postmenopausal osteoporosis. We also performed RNA sequencing to show that the PI3K/AKT signaling pathway and oxidation-reduction reaction were essentially involved in the osteogenic process induced by metformin.

In previous studies, there was no consistent conclusion regarding the pathogenesis of postmenopausal osteoporosis. Our transcriptional data indicated that metformin modulated the expression of genes involved in oxidation-reduction process of osteoblasts. We also believe that oxidation-reduction imbalance is a key factor leading to osteoporosis in postmenopausal women due to the reducibility of estrogen. It has been demonstrated that circulating levels of catalase, superoxide dismutase 2 (SOD 2) and peroxiredoxin 2 (PRX2) are lower in postmenopausal women with osteoporosis than health controls (Azizieh et al., 2019). Mitochondrial dysfunction leads to weakened internal oxidoreductase function, resulting in high levels of free radical, which attacks osteoblasts and induces apoptosis (Zhou et al., 2019). Osteoclasts showed strong differentiation when the inhibitory effect of estrogen was lost (Li et al., 2021). Metformin can improve the oxidative state and protect against oxidative stress damage, which also indicates the therapeutic effect of metformin on osteoporosis (Jia et al., 2021). Therefore, it is essential to determine the mechanism by which metformin acts on the oxidation-reduction process in osteoblasts.

**FIGURE 5:**
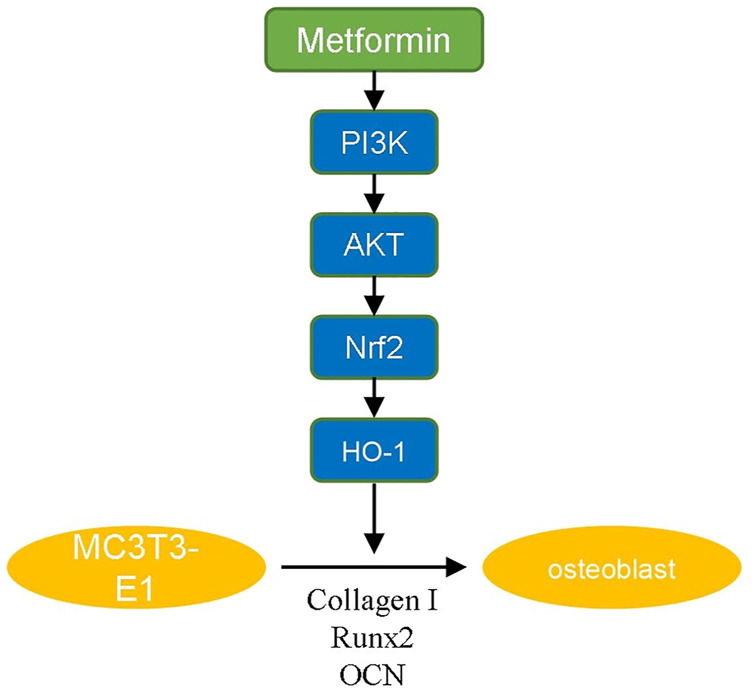
The summarized figure of signaling pathway.

PI3K/AKT signaling is an important pathway involved in the modulation of redox balance. Activation of PI3K/AKT enhances the antioxidant effect (Kim et al., 2021; Wang P et al., 2021). The PI3K/AKT signaling pathway has been demonstrated to have a positive effect on osteoblast differentiation and inhibition of PI3K/AKT signaling suppresses the osteoinduction process (Zhang et al., 2019; Dong et al., 2020). It was reported that multiple drugs promote osteogenic differentiation *via* the PI3K/AKT pathway (Xiong et al., 2020; Liu et al., 2021). In our study, we revealed that metformin promoted osteogenic differentiation of MC3T3-E1 cells by activating the PI3K/AKT pathway. Various osteogenic growth factors and extracellular matrix components can induce the activation of PI3K, including fibroblast growth factor, vascular endothelial growth factor, angiogenic protein I and insulin (Khodabandehloo et al., 2020). These factors activate receptor tyrosine kinases, cause autophosphorylation and induce PI3K/AKT-mediated differentiation. We also demonstrated that the osteogenic effect of metformin was blocked by adding a PI3K/AKT inhibitor. The inhibitor decreased the ratio of OPG/RANKL expression to inhibit osteogenesis (Xu et al., 2021). Additionally, we used H_2_O_2_ to mimic the oxidative damage of osteoblasts metformin to attenuate it, the anti-apoptosis effect of which has been proven in our previous study (Yang et al., 2021). And in this study, we further explored the downstream mechanism. Nrf2 is an factor involved in the modulation of cellular oxidation-reduction balance (Čipak Gašparović et al., 2021). Nrf2 participates in the expression and maturation of various anti-oxidative proteins to maintain the homeostasis of oxidation and reduction (Ying et al., 2018). HO-1 is an essential anti-oxidative factor modulated by Nrf2 (Ma et al., 2021). HO-1 decomposes heme to produce carbon monoxide (CO) that promotes the expression of glutamate-cysteine ligase for GSH transformation (Consoli et al., 2021). As mentioned above, PI3K/AKT/Nrf2/HO-1 pathway is closely related to osteogenic differentiation and anti-oxidative damage of osteoblasts. Our experimental results also indicated protective effect of metformin in postmenopausal osteoporosis *via* this pathway (Figure 5).

Due to the complex pathogenesis of osteoporosis, current treatment methods have limited efficacy. According to our experiments, metformin can directly increase the gene expression, protein formation of osteogenic markers and protect H_2_O_2_-induced oxidative damage. *In vitro*, we further verified that the bone mass of the OVX mice was significantly improved by metformin feeding. As estrogen is a reductive hormone, detecting the role of oxidoreduction in osteoporosis development and the therapeutic effect of metformin in improving the oxidative state in osteoblasts will be further directions for research on postmenopausal osteoporosis.

## Conclusion

Our study demonstrated that metformin could promote osteogenic differentiation and improve H_2_O_2_-induced oxidative damage of osteoblasts *via* the PI3K/AKT/Nrf2/HO-1 pathway. Vitro experiments also demonstrated that metformin improved bone mass, enhanced osteogenic protein expression and increased anti-oxidative level in OVX mice. These results provide an important basis for the potential therapeutic effect of metformin in postmenopausal osteoporosis. Along with its effect in controlling blood glucose and reducing lipids, metformin may have applications in the treatment of osteoporosis.

## Data Availability

The data presented in the study are deposited in the GEO (NCBI) repository, accession number (GSE198254). https://www.ncbi.nlm.nih.gov/geo/query/acc.cgi?acc=GSE198254/Supplementary Material.
