# Supplementary figures and images for: Metformin Promotes Differentiation and Attenuates H2O2-Induced Oxidative Damage of Osteoblasts via the PI3K/AKT/Nrf2/HO-1 Pathway
Source: Front Pharmacol. 2022 Mar 21;13:829830. doi: 10.3389/fphar.2022.829830 (PMC8978328; doi:10.3389/fphar.2022.829830)

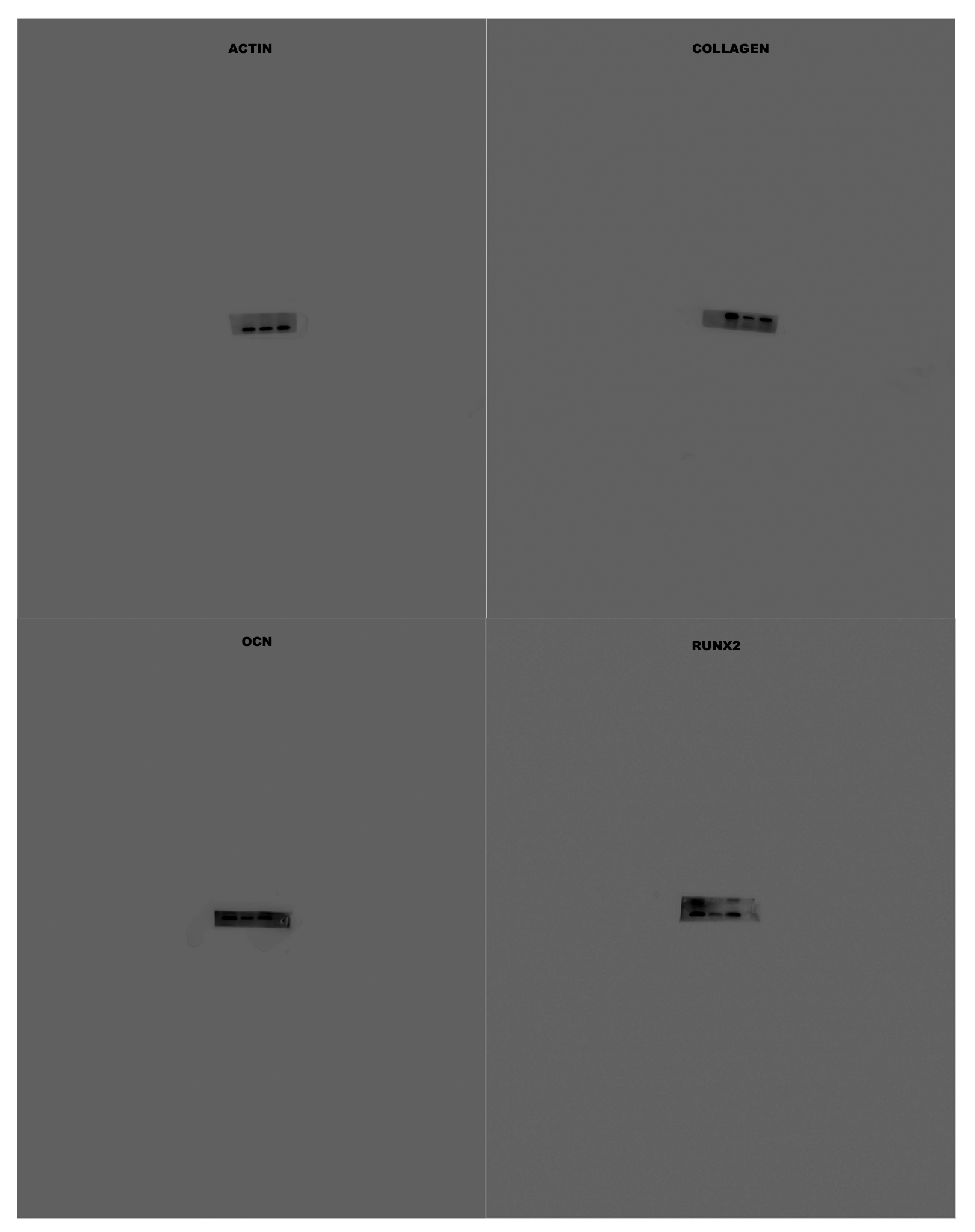

Supplement: Supplementary file 1 [file Image3.JPEG]

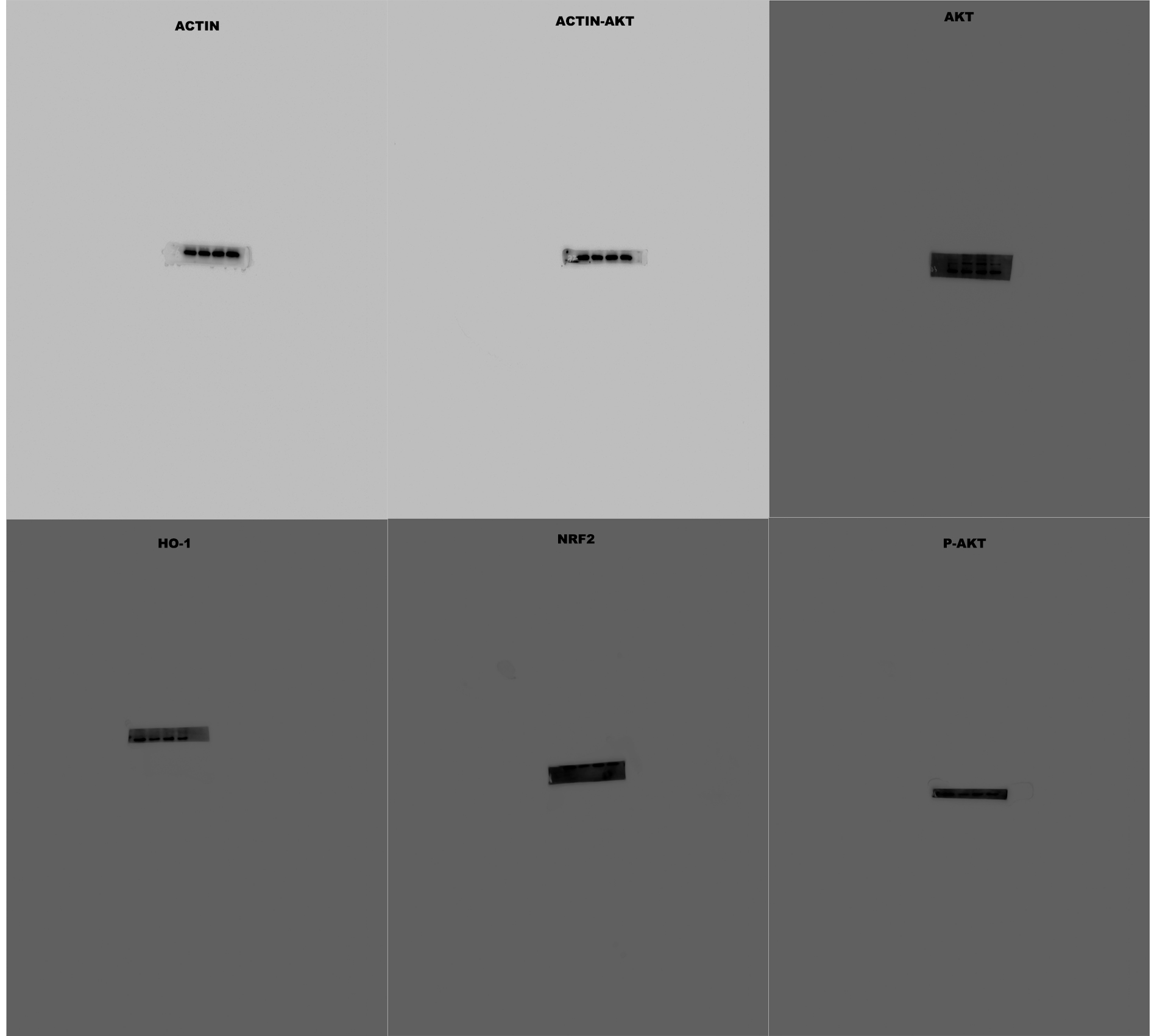

Supplement: Supplementary file 3 [file Image1.JPEG]

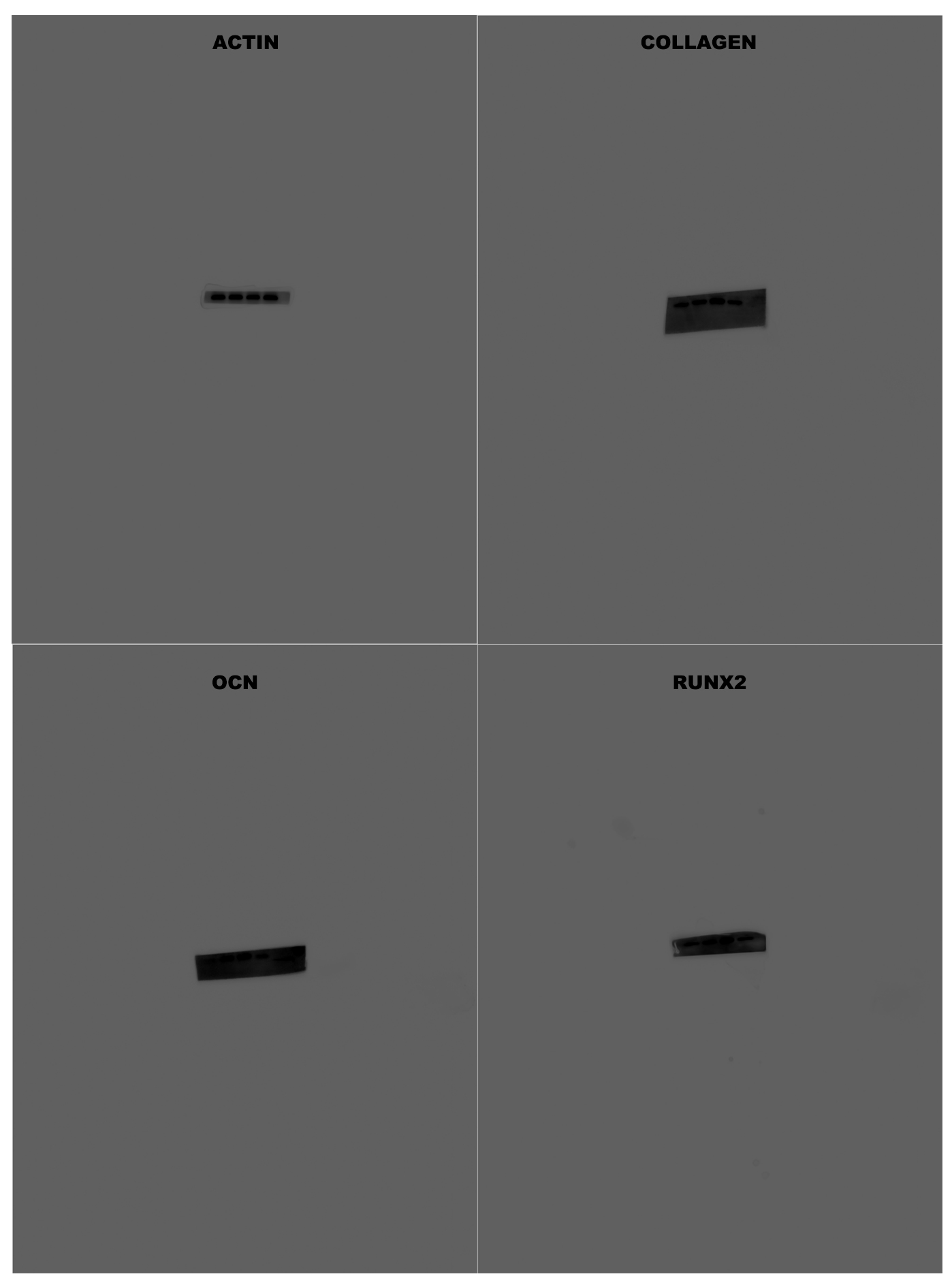

Supplement: Supplementary file 4 [file Image4.JPEG]

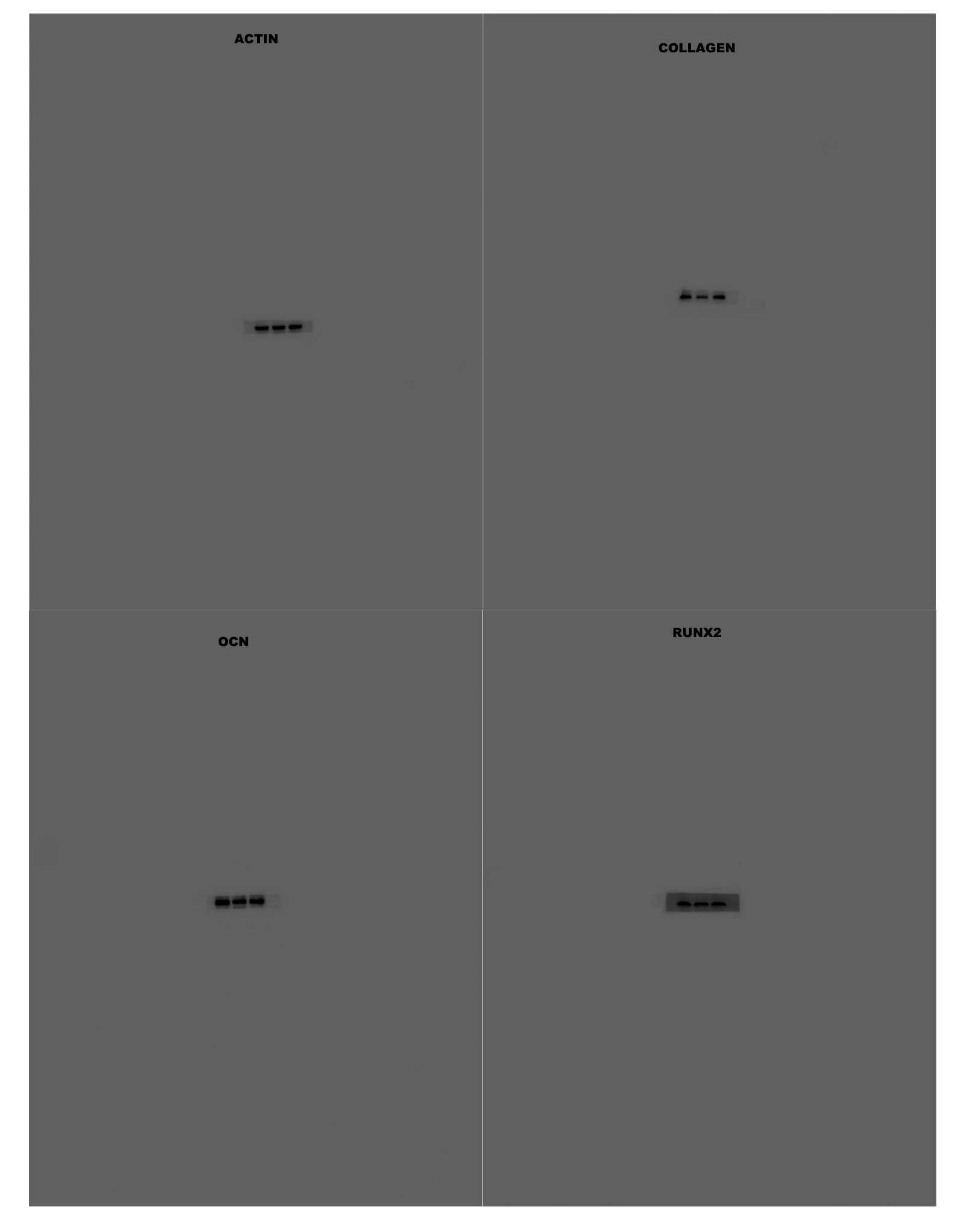

Supplement: Supplementary file 5 [file Image2.JPEG]

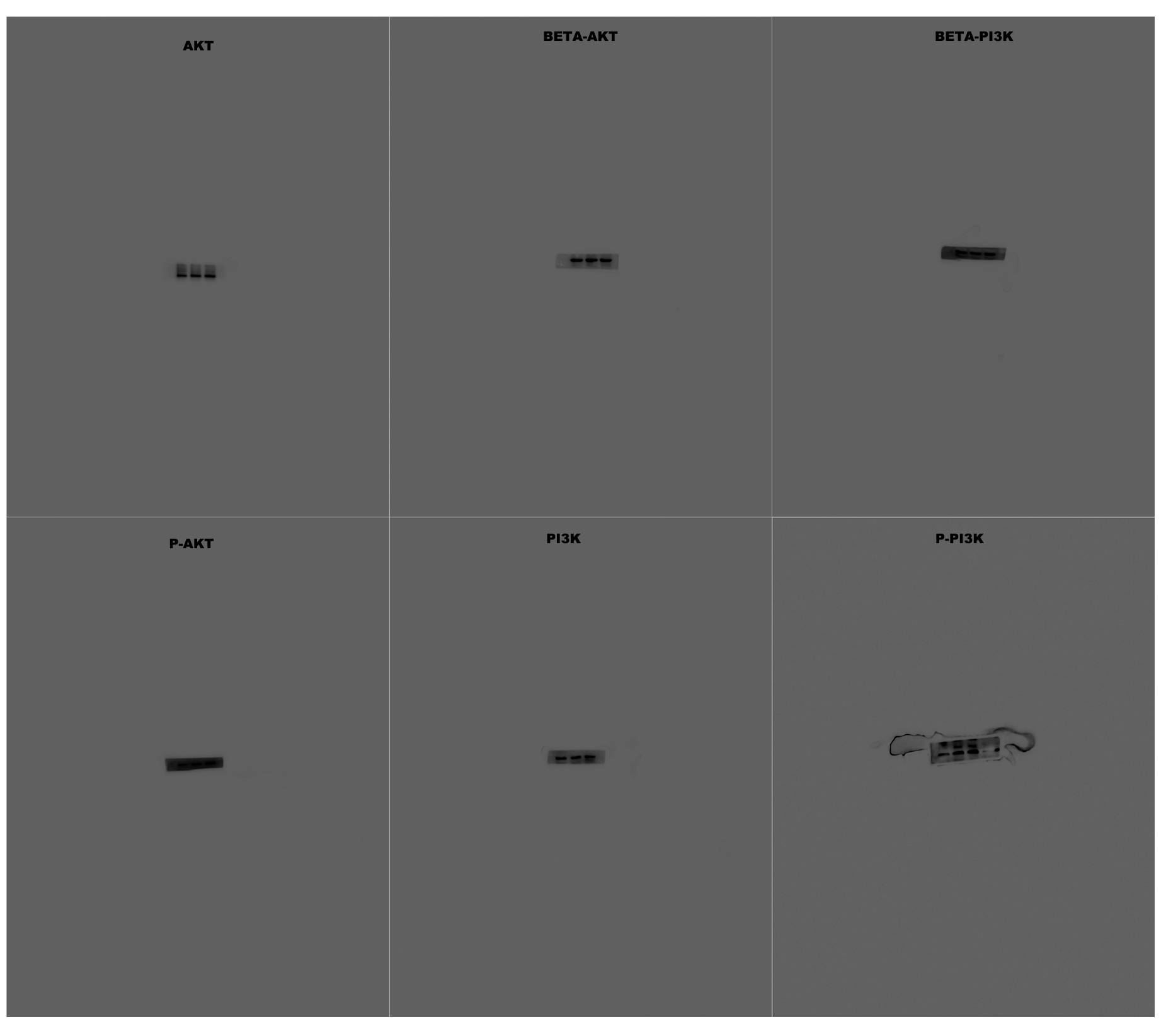

Supplement: Supplementary file 6 [file Image5.JPEG]

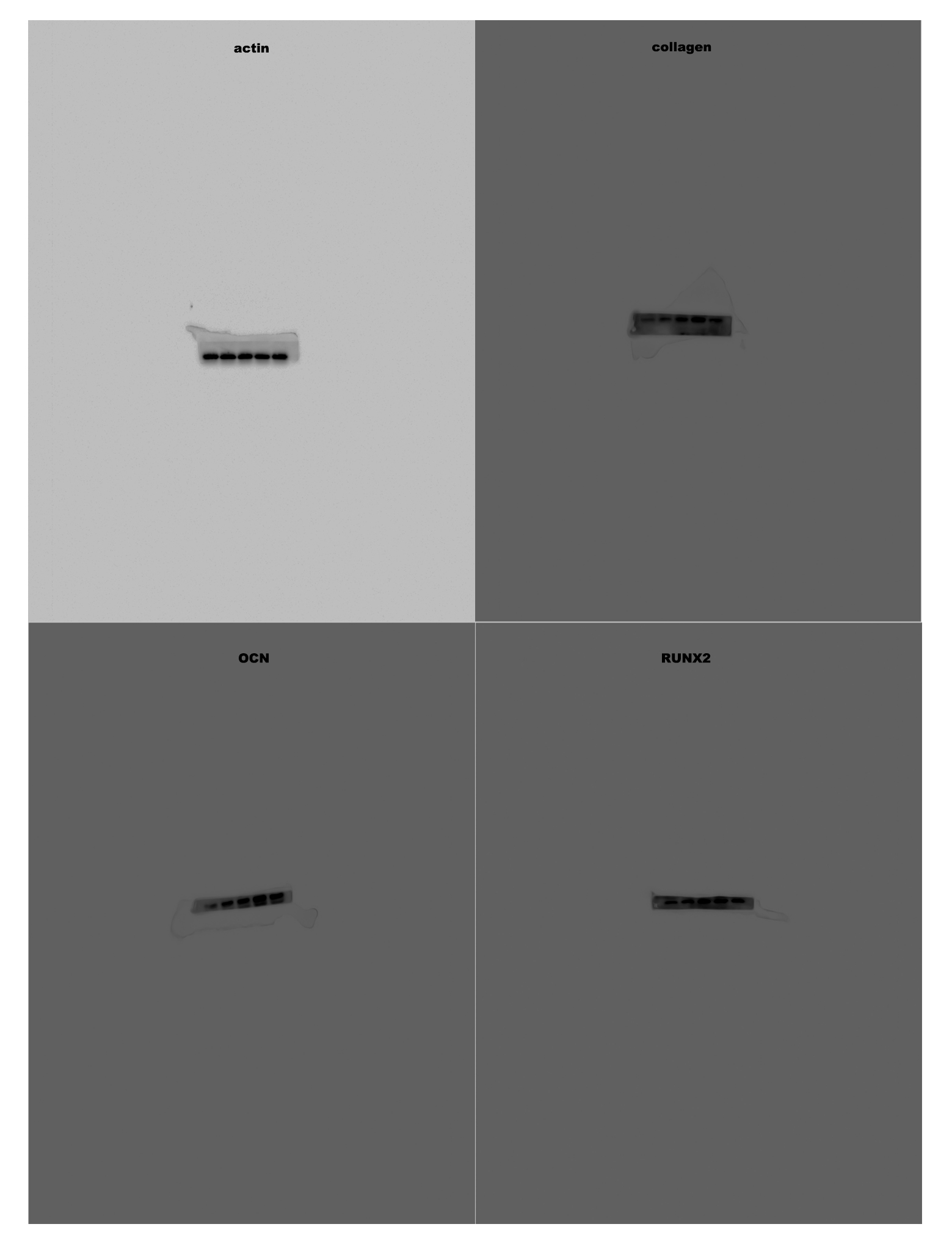

Supplement: Supplementary file 7 [file Image6.JPEG]
