# Supplementary material for: Metformin Promotes Differentiation and Attenuates H2O2-Induced Oxidative Damage of Osteoblasts via the PI3K/AKT/Nrf2/HO-1 Pathway
Source: Front Pharmacol. 2022 Mar 21;13:829830. doi: 10.3389/fphar.2022.829830 (PMC8978328; doi:10.3389/fphar.2022.829830)
Supplement: Supplementary file 2 [file Table1.DOCX]

| Genes | Forward(5'-3') | Reserve(5'-3') |
| --- | --- | --- |
| COLL-1 | ACCTCCCAGTGGCGGTTATGAC | AGTTCTTCTGAGGCACAGACGG |
| OCN | CTCCCATTGGCGAGTTTG | TGTAGTCCAGGTGGAGCTTGTG |
| Runx2 | TTTGCAGTGGGACCGACA | AGCCATGGTGCCCGTTAG |
| β-Actin | GGCTGTATTCCCCTCCATCG | CCAGTTGGTAACAATGCCATGT |
